# Psychiatric comorbidity among patients attending an addiction treatment center in Morocco

**DOI:** 10.1192/j.eurpsy.2022.2157

**Published:** 2022-09-01

**Authors:** A. Jelti, F. Kennab, F. N’Sabi, B. Oneib, F. Elghazouani

**Affiliations:** Mohammed VI University Hospital, Faculty of Medicine and Pharmacy, University Mohammed Premier, Department Of Psychiatry, Oujda, Morocco

**Keywords:** addictive disorders, Psychiatric comorbidity, dual diagnosis

## Abstract

**Introduction:**

The comorbidity between psychiatric disorders and substance use disorders is more and more common in daily clinical practice. However, only few studies have adressed this subject in north african patients.

**Objectives:**

The main objective of our study was the estimation of the prevalence and patterns of psychiatric co-morbidities in substance users seeking care.

**Methods:**

Our work consisted of a cross-sectional study of a sample of patients attending outpatient substance use treatment at the addiction center in Oujda, Morocco. A hetero-questionnaire was used to collect sociodemographic data and patient history, DSM-IV criteria to assess substance abuse and dependence, and the Mini-International Neuropsychiatric Interview [MINI] to assess psychiatric comorbidities.

**Results:**

Our study involved 100 patients, with a male predominance (89% of users). The main substances used in the last 12 months were tobacco (78%), followed by cannabis (74%), alcohol (50%), and benzodiazepines (44%). Psychiatric comorbidity was identified in 71% of the users, 51% of whom had a depressive disorder, 35% an anxiety disorder and 10% a gambling disorder. The dependence on the substance that initially motivated the consultation was higher in patients with psychiatric comorbidity (p=0.033). The post-traumatic stress disorder was significantly associated with the presence of alcohol dependence (p=0.028). The presence of benzodiazepine dependence (p=0.025) and abuse of cocaine (p=0.028) and Ecstasy (p=0.000) were significantly associated with suicide risk.

**Conclusions:**

Our study found a high prevalence of psychiatric comorbidities among substance users seeking treatment, this should prompt clinicians to pay particular attention to this issue in order to adapt and improve their management.

**Disclosure:**

No significant relationships.

